# Developmental Change in Cognitive Emotion Regulation Profiles in the Transition from Childhood to Adolescence

**DOI:** 10.1007/s10802-025-01375-1

**Published:** 2025-10-07

**Authors:** Annemiek Karreman, Elisabeth L. de Moor, Odilia M. Laceulle

**Affiliations:** 1 Department of Medical and Clinical Psychology, CoRPS – Center of Research on Psychological Disorders and Somatic Diseases, Tilburg University, Warandelaan 2, 5037 AB Tilburg, the Netherlands; 2 Department of Developmental Psychology, Tilburg University, Tilburg, the Netherlands; 3 Department of Developmental Psychology, Utrecht University, Utrecht, the Netherlands

**Keywords:** Cognitive emotion regulation profiles, Latent profile analysis, Latent profile transition analysis, Internalizing problems, Youth

## Abstract

A person-centered approach studying cognitive emotion regulation profiles rather than single strategies can shed light on adaptive and maladaptive cognitive emotion regulation in youths. This study extends previous research by examining self-reported cognitive emotion regulation profiles not only cross-sectionally but also longitudinally, in the transition from childhood to adolescence. To examine the adaptive or maladaptive nature of the profiles, cross-sectional associations with parent-reported internalizing problems were tested in a subsample. Data came from a 3-wave longitudinal project on Dutch youths (Wave 1: *N* = 526, mean age = 10.1 years). Youths and parents completed questionnaires yearly. Latent Profile Analyses revealed three profiles at each wave: (1) a “Generally low” profile, consisting of little use of all cognitive emotion regulation strategies, (2) a “High adaptive, low maladaptive” profile, and (3) a “Low adaptive, high maladaptive” profile. Latent Profile Transition Analyses showed substantial stability in profiles over time but also transitions; most towards a less adaptive profile, some towards a more adaptive profile. The maladaptive nature of the third profile was confirmed by cross-sectional associations with parent-reported internalizing problems (at Wave 1 and 2 but not Wave 3). The results showing profile transitions in some youths may yield insights into the development of cognitive emotion regulation abilities.

**Supplementary Information:**

The online version contains supplementary material available at 10.1007/s10802-025-01375-1.

Cognitive emotion regulation abilities are important to effectively manage emotions after stressful experiences (Garnefski et al., 2001). The transition from childhood to adolescence is a critical period for developing these abilities, with youths being at risk of dysfunctional emotion regulation (Casey et al., 2010). While most research has focused on single emotion regulation strategies and their associations with psychopathology (Compas et al., 2017; Schäfer et al., 2017), recent cross-sectional studies show that emotion regulation profiles can be identified based on the type and extent of emotion regulation strategy use (i.e., van den Heuvel et al., 2020). This shift from a variable- to a person-centered approach offers new insight into emotion regulation. However, longitudinal research is needed to investigate the developmental course of emotion regulation profiles. The current study investigates cognitive emotion regulation profiles cross-sectionally and longitudinally from ages 10 to 12 and examines the adaptive or maladaptive nature of the profiles by testing cross-sectional associations with parent-reported internalizing problems.

## Cognitive Emotion Regulation

Cognitive emotion regulation refers to the conscious modification of emotionally arousing information (Garnefski et al., 2001). Cognitive strategies, such as positive reappraisal and putting into perspective, are primarily used to influence the emotion generation process at the appraisal stage, by changing how the situation is viewed in light of motivational concerns. However, some strategies (e.g., acceptance) may also be used later in the process to modulate the emotional response (Gross et al., 2019). Strategies can be adaptive or maladaptive in the moment, depending on whether their use is synchronized with contextual demands and facilitates goal pursuit (Aldao et al., 2015; Bonanno & Burton, 2013). However, based on the habitual use of cognitive strategies across contexts, specific strategies have been labeled adaptive (i.e., acceptance, refocus on planning, positive refocusing, positive reappraisal, putting into perspective) or maladaptive (i.e., self-blame, other-blame, rumination, catastrophizing) as their use has been consistently associated with internalizing problems, such as depressive symptoms and anxiety (e.g., Garnefski et al., 2001; Garnefski & Kraaij, 2006b; Legerstee et al., 2010)^1^.

When children enter adolescence, their cognitive emotion regulation abilities undergo notable development. Developmental theory suggests that emotion regulation skills that young children have internalized continue to develop (Bariola et al., 2011; Zeman et al., 2006). As prefrontal regions mature from childhood to adulthood, cognitive abilities improve, related to improved cognitive emotion regulation (Casey et al., 2010; Crone & Dahl, 2012; Ochsner & Gross, 2008; Somerville et al., 2010). However, hormonal and neurodevelopmental changes, starting at the onset of puberty, cause increased emotional reactivity. Adolescents’ pursuit of autonomy and societal expectations that adolescents independently regulate emotions can also result in intense and fluctuating emotions (Calkins & Mackler, 2011). Heightened emotional reactivity is thought to deplete cognitive control, which can lead to dysfunctional emotion regulation, with the risk of developing internalizing problems (Casey et al., 2010; Zeman et al., 2006). Adolescents with internalizing problems appear to have a more cognitive than behavioral regulation style, employing more cognitive than behavioral (e.g., distraction) emotion regulation strategies (te Brinke et al., 2021). This makes the study of cognitive emotion regulation during the transition from childhood to adolescence particularly important.

## Variable- Versus Person-Centered Approach

Studies focusing on single emotion regulation strategies (i.e., variable-centered approach) reveal important information about how each strategy is associated with internalizing problems (e.g., Garnefski et al., 2001; Garnefski & Kraaij, 2006b; Legerstee et al., 2010), but do not capture the entire process of habitual emotion regulation. That is, they do not consider individuals’ emotion regulation repertoire, defined as the range of emotion regulation strategies that youths can use to meet diverse contextual demands (Bonanno & Burton, 2013). An extensive repertoire is suggested to help flexibly regulate emotions and therefore to be more adaptive than a narrow repertoire, as there are more options to choose from when selecting an emotion regulation strategy. The composition of the repertoire (i.e., the specific strategies that make up the repertoire) is also important because an extensive repertoire consisting only of strategies that have been established as maladaptive does not help to flexibly regulate emotions (Aldao et al., 2015; Bonanno & Burton, 2013). Due to its focus on the use of isolated emotion regulation strategies, the variable-centered approach does not capture the constellation of different emotion regulation strategies used within a person.

A person-centered approach, such as Latent Profile Analysis (LPA), focuses on patterns of strategy use within individuals to capture essential features of emotion regulation and therefore sheds more light on the holistic use of emotion regulation strategies than the variable-centered approach. LPA identifies profiles of scores on different emotion regulation strategies and estimates the probability that individuals are in each of the profiles (Vermunt et al., 2008). These probabilities can be linked to other constructs, such as adjustment. This way, groups of individuals with maladaptive profiles can be distinguished from groups of individuals with more normative profiles. Studying youths’ cognitive emotion regulation profiles in explaining internalizing problems thus has the potential to elucidate the habitual use of combinations of cognitive emotion regulation strategies within individuals that may be clinically relevant.

Cross-sectional studies that applied a person-centered approach provided support for qualitatively and quantitatively different emotion regulation profiles in adults (Chesney & Gordon, 2017; Chesney et al., 2019; De France & Hollenstein, 2017; Dixon-Gordon et al., 2015; Eftekhari et al., 2009) and adolescents (Lougheed & Hollenstein, 2012; van den Heuvel et al., 2020). Studies primarily examined a diversity of cognitive and behavioral emotion regulation strategies. Van den Heuvel and colleagues (2020) were the only researchers focusing exclusively on cognitive emotion regulation profiles in adolescents (aged 12 to 21 years, all having experienced a stressful life event). They identified four profiles, indicating adolescents who made little use of all strategies (10%), much use of all strategies (44%), much use of maladaptive strategies and little use of adaptive strategies (12%), and medium use of adaptive strategies (except a below average use of acceptance and refocus on planning) and below average use of maladaptive strategies (34%). The number of profiles identified in other studies ranges from three to six (e.g., Chesney et al., 2019; De France & Hollenstein, 2017; Lougheed & Hollenstein, 2012). In general, profiles have been identified based on (1) the range of strategies used by individuals (low to high range), indicating the size of the emotion regulation repertoire, and (2) the use of strategies established as adaptive or maladaptive, indicating the combinations of strategies in individuals’ emotion regulation repertoire.

To better understand the adaptive or maladaptive nature of cognitive emotion regulation profiles it is relevant to know how profiles are associated with internalizing problems. Cross-sectional person-centered studies on adolescents and young adults suggest that particularly the combinations of strategies in individuals’ emotion regulation repertoire are markers of adjustment. Studies consistently show that profiles with much use of maladaptive strategies and little use of adaptive strategies were associated with higher levels of internalizing problems (Chesney & Gordon, 2017; Chesney et al., 2019; De France & Hollenstein, 2017; Dixon-Gordon et al., 2015; van den Heuvel et al., 2020). Profiles with much use of adaptive strategies and little use of maladaptive strategies were associated with lower levels of internalizing problems in most adult studies (Chesney & Gordon, 2017; Chesney et al., 2019; Dixon-Gordon et al., 2015; Eftekhari et al., 2009).

There is less consensus about the relevance of the size of the emotion regulation repertoire for adjustment. Adolescents with profiles indicating a narrow range of strategies reported more internalizing problems than those with profiles indicating a broader range of strategies in one study (Lougheed & Hollenstein, 2012), but not in another study (van den Heuvel et al., 2020). Dixon-Gordon and colleagues (2015) found in undergraduate students that those who reported little use of all strategies showed fewer anxiety symptoms than those who reported much use of all strategies and a relatively high use of maladaptive strategies (i.e., worrying, rumination). Overall, the study findings suggest that an extensive repertoire of strategies, considered to help flexibly regulate emotions (Aldao et al., 2015; Bonanno & Burton, 2013), is not so much of importance in relation to internalizing problems. Rather, the combinations of strategies in the repertoire—in adolescents, particularly the high use of maladaptive strategies and low use of adaptive strategies—seem to matter most in the context of internalizing problems.

## Change in Cognitive Emotion Regulation Profiles from Childhood to Adolescence

Theories on habitual emotion regulation conceptualize emotion regulation strategies as dispositional characteristics with high rank-order stability (Gross & John, 2003; Liu & Thompson, 2017). Developmental theory further suggests normative mean-level change as emotion regulation skills continue to develop from childhood to adolescence (Bariola et al., 2011; Zeman et al., 2006). Therefore, based on theory we might expect cognitive emotion regulation profiles to be largely stable over time. However, longitudinal research is needed to understand normative stability and changes in profiles and the development of profiles. Latent Profile Transition Analysis (LPTA) estimates the probability that individuals remain in the same profile or transition from one profile to another over time (Vermunt et al., 2008). This way, we can identify groups of individuals who are at risk of remaining in maladaptive profiles or tend to move towards less adaptive profiles. Although theory suggests stability in profiles, the transition from childhood to adolescence may be a sensitive period for transitions in cognitive emotion regulation profiles. There may be a risk of transitioning to a less adaptive profile due to pubertal biological and psychosocial changes causing heightened emotional reactivity and vulnerability in emotion regulation (Casey et al., 2010; Crone & Dahl, 2012; Somerville et al., 2010). To our knowledge, no study has longitudinally examined emotion regulation profiles to investigate developmental changes in profiles over time.

Though not examined directly, previous cross-sectional person-centered research suggests some normative change in emotion regulation profiles in adolescence. For instance, van den Heuvel and colleagues (2020) found in their sample of 12- to 21-year-old adolescents that the adolescents in the profile indicating infrequent use of both adaptive and maladaptive cognitive emotion regulation strategies (10% of their sample) were relatively young. In contrast, Chesney and Gordon (2017) did not identify a group of participants who infrequently used all cognitive and behavioral strategies in their adult community sample. These results may imply a developmental trend towards profiles with more frequent use of emotion regulation strategies (i.e., a more extensive emotion regulation repertoire) in early adolescence.

Variable-centered studies further inform hypotheses on profile transitions. Consistent with theory (Bariola et al., 2011; Zeman et al., 2006), research shows that emotion regulation abilities increase across adolescence, especially in adolescents with initially lower abilities (Herd et al., 2020). Longitudinal studies focusing exclusively on the strategy rumination show moderate rank-order stability in adolescence, with inconsistent findings on mean-level stability (Hankin, 2008; Mazzer et al., 2019). A study on cognitive reappraisal and expressive suppression reports no mean-level change from middle to late adolescence (Herd et al., 2022). Cross-sectional variable-centered studies including multiple strategies suggest a trend towards less adaptive and more maladaptive strategy use in middle adolescence (Cracco et al., 2017; Gullone et al., 2010; Zimmermann & Iwanski, 2014). Additionally, older adolescents (16 − 18) use emotion regulation strategies more frequently than younger adolescents (12 − 15) (Garnefski & Kraaij, 2006b). Thus, transitions in cognitive emotion regulation profiles could be expected, particularly in the direction of more frequent use of strategies and possibly in the direction of more maladaptive and less adaptive profiles. However, longitudinal studies examining combined strategy use over time, especially in the transition from childhood to adolescence, are lacking.

## The Current Study

The aim of the current study was to investigate cognitive emotion regulation profiles cross-sectionally and longitudinally in the developmental transition from childhood to adolescence. First, we cross-sectionally examined which cognitive emotion regulation profiles can be identified as patterns of cognitive emotion regulation strategies at the ages of 10, 11, and 12 years. We expected to identify profiles showing less use and more use of all cognitive emotion regulation strategies relative to the sample mean. We also expected to identify distinct profiles with specific combinations of cognitive emotion regulation strategies: more use of strategies established as adaptive and less use of strategies established as maladaptive, and less use of strategies established as adaptive and more use of strategies established as maladaptive (all relative to the sample mean), respectively. Second, we longitudinally investigated the extent to which youths remain stable in, or transition between, cognitive emotion regulation profiles across three annual measurement points (ages 10 − 12). We expected the majority of youths to remain stable in their profiles. A smaller proportion is expected to transition from one profile to another, with most of these youths expected to transition in the direction of more frequent use of all emotion regulation strategies.

In addition, we tested cross-sectional^2^ associations with parent-reported internalizing problems in a subsample. We expected that the adaptive or maladaptive nature of profiles would be confirmed by associations with internalizing problems. Notably, most previous studies relied on self-report only, which may have introduced bias (e.g., Chesney et al., 2019; Dixon-Gordon et al., 2015; van den Heuvel et al., 2020). Therefore, to reduce shared method bias, we aimed to confirm the associations using parent reports of internalizing problems, as youths provided the emotion regulation ratings. No associations were expected between the profiles indicating the size of the emotion regulation repertoire and internalizing problems.

## Method

We report how we determined our sample size, all data exclusions, all manipulations, and all measures in the study.

### Participants and Procedure

The data for this study are part of a longitudinal research project on vulnerability and resilience in the transition from childhood to adolescence. A total of 536 participants were recruited, 10 of whom were excluded because they lacked cognitive emotion regulation or internalizing problem data in all waves. The resulting study sample consisted of 526 participants (*M*_age at Wave 1_ = 10.1, *SD* = 0.45), of whom 235 (47.5%) identified as a girl. Of the participating parents (*n* = 383), 322 (84.1%) identified as a woman. Most participating parents (*n* = 226) and their partners had an average education level of higher vocational education (equivalent of college education, with a professional orientation) or higher (40.4%).

Youths were recruited at 24 primary schools in the Netherlands. Data were collected by research assistants in three annual waves (Wave 1–Wave 3) from 2017 to 2020. All measurement occasions were in the second half of the school year, with the first measurement occasion taking place in the second half of the sixth year (equivalent to fourth grade). Participating youths completed questionnaires in the classroom setting, using the computer or pen and paper. Participating parents completed questionnaires at home on their computers.

After schools confirmed their participation, written informed consent was obtained from teachers and parents at each wave. Youths gave assent at each wave. Teachers distributed information letters to parents in the first wave, while research assistants directly approached the parents of participating children in the second and third waves. Participation was thus initiated by teachers to a greater extent in the first wave than in subsequent waves, which may partially explain substantial dropout across waves (59.7% of participants were still participating at Wave 2 and 42 0.2% at Wave 3). The sample size was determined for the larger research project (planned *N* = 500) and not specific for the current study. This study received ethical approval from the Ethics Review Board of the School of Social and Behavioral Sciences, Tilburg University (protocol number: EC-2016.63).

### Measurement Instruments

#### Cognitive Emotion Regulation Strategies

In each wave, youths completed the short version of the Cognitive Emotion Regulation Questionnaire–kids version (CERQ-k; Garnefski et al., 2007) for the assessment of cognitive emotion regulation strategies. This version, based on the 18-item version for adults (Garnefski & Kraaij, 2006a), consists of nine two-item subscales: self-blame, other-blame, acceptance, refocus on planning, positive refocusing, rumination, positive reappraisal, putting into perspective, and catastrophizing. Youths were asked to indicate what they usually think when they experience something unpleasant. They rated items (e.g., “I think that I can learn from it”) on a 5-point Likert scale from 1 (almost never) to 5 (almost always). Higher scores indicate more use of the cognitive emotion regulation strategy.

Previous research on the reliability and validity suggested acceptable reliability (i.e., Cronbach’s alphas 0.62 − 0.79) and supported criterion validity (i.e., in relation to depression and anxiety measures) when it comes to the original CERQ-k subscales (Garnefski et al., 2007). With regard to the subscales of the short version of the CERQ-k, reliability is inherently challenged given the 2-item nature of these subscales. The reliability of the 2-item subscales varies considerably across studies (e.g., ordinal alphas 0.47 − 0.70 for short Spanish version of the CERQ-k [Orgilés et al., 2019], Cronbach’s alphas 0.67 − 0.81 for CERQ-short for adults [Garnefski & Kraaij, 2006a]). In the current study, Spearman-Brown coefficients (Eisinga et al., 2013) were 0.50 − 0.79 (average 0.64) at Wave 1, 0.54 − 0.81 (average 0.66) at Wave 2, and 0.47 − 0.86 (average 0.73) at Wave 3.

#### Internalizing Problems

In each wave, parents completed the Dutch translation of the Child Behavior Checklist for ages 6–18 (CBCL/6–18; Achenbach & Rescorla, 2001; Verhulst & Van der Ende, 2013). This study used the broad-band scale of internalizing problems, consisting of the syndrome scales Anxious/Depressed (13 items), Withdrawn/Depressed (8 items), and Somatic Complaints (12 items). Parents rated items to evaluate their children’s emotional problems (e.g., “Nervous, high-strung, or tense”) on a 3-point Likert scale (1 = not true, 2 = somewhat or sometimes true, 3 = very true or often true). Internalizing problems raw scores were used, with higher scores indicating more internalizing problems. Psychometric properties of the Dutch translation are adequate (Verhulst & Van der Ende, 2013). Studies indicate that the CBCL is an efficient screening tool for detecting psychiatric disorders in youth (Warnick et al., 2008). In the current study, the internalizing problems scale showed acceptable reliability at all three waves (ω_total_ = 0.96, 0.98, and 0.95, respectively, excluding item 81 on Wave 3 due to a lack of variation^3^).

### Statistical Analysis Plan

#### Preparatory Analyses

Before turning to the main analyses to test our hypotheses, we first examined missingness and dropout in our data (see Supplemental Materials). Next, because the participants in our sample were recruited through schools, we also tested for interdependency within schools by calculating intraclass correlations (ICC) and design effects (DE) for the baseline mean scores of the nine cognitive emotion regulation strategies. The ICCs and DEs were all between − 0.02 and 0.05 and 0.65–1.93 respectively, indicating that there was no substantial clustering in our data based on participants’ school (Muthén & Satorra, 1995). The exception to this was internalizing problems, for which the DE exceeded the critical score of 2 (ICC = 0.17, DE = 4.31). We controlled for the design effect in analyses including internalizing problems. Finally, we checked the structure of the internalizing problems scale by estimating one-factor CFA and examined whether this structure was invariant across time (see Supplemental Materials).

#### Main Analyses

##### Clustering of the Cognitive Emotion Regulation Strategies

To examine the manifestation of cognitive emotion regulation strategies in youth and how this manifestation changes over time, we fit a series of LPAs and LPTAs. The number of indicators affects the statistical power of LPA (Tein et al., 2013). In our models, we included mean scores on each of the nine cognitive emotion regulation strategies assessed by the CERQ-k (Garnefski et al., 2007) as indicators for the profiles. Each of these strategies reflects the conscious cognitive processing of emotionally arousing information and is linked to internalizing problems (e.g., Garnefski et al., 2001; Garnefski & Kraaij, 2006b; Legerstee et al., 2010). Including all strategies is essential to test our hypotheses about cognitive emotion regulation profiles, which vary in the extent and combination of strategy use. This approach aligns with theory on the emotion regulation repertoire, emphasizing the importance of the flexible use of multiple strategies (Aldao et al., 2015; Bonanno & Burton, 2013) and ensures comparability with prior research focused on cognitive emotion regulation (e.g., van den Heuvel et al., 2020). We did not perform an a-priori power analysis for the current study, as this is complex due to the fact that statistical power depends on several factors, such as the sample size, number of indicators, and the actual number of profiles, which is not known in advance (Tein et al., 2013).

To identify the optimal, most parsimonious model solution in the LPA that best captures patterns in our data, we estimated solutions with one to five profiles for each wave separately. We did not estimate LPA solutions with six or more profiles for each wave to ensure enough power considering our sample size (Tein et al., 2013). We determined the best-fitting solution based on the Bayesian Information Criterium (BIC), the sample-size adjusted Bayesian Information Criterium (SSA-BIC), the Vuong-Lo-Mendell-Rubin Likelihood Ratio Test (VLMR-LRT), the entropy, and the average classification probabilities for the most likely class (ACPMLC). Specifically, we favored model solutions with lower BIC and SSA-BIC values, as these indicate better model fit, and higher entropy and ACPMLC values, as these indicate higher clarity and accuracy of classification. We used a cutoff of 0.80 for entropy and 0.70 for ACPMLC (Clark & Muthén, 2009; Nylund-Gibson & Choi, 2018). For the VLMR-LRT, where a significant *p* value indicates an improvement in model fit from a model with *k* – 1 profiles to the model with *k* profiles, we preferred models with a *p* < .05. In addition to these statistical criteria, we determined that profiles should be clearly distinguishable, conceptually meaningful (i.e., substantially different from other profiles) and should contain at least 5% of the sample to ensure practical meaningfulness. After selecting the optimal model solution for the LPA at each wave, we added child gender as predictor to the model following the 3-step method (R3STEP), so that this predictor did not alter specification of the model (Asparouhov & Muthén, 2014).

Next, we fit an LPTA for all waves together. To ensure the robustness of this solution, we estimated (a) LPTAs with *k* – 1 profiles, *k* profiles, and *k* + 1 profiles, and (b) in addition to an LPTA that was fully invariant across waves (i.e., constrained means of the cognitive emotion regulation strategies across time) also an LPTA that was fully noninvariant (i.e., all means of the cognitive emotion regulation strategies were freely estimated across time). In case the fully invariant model fit worse than the fully noninvariant model, we additionally fit a model of partial invariance, in which some means were freely estimated, based on the fully noninvariant model. Change in model fit was assessed with the BIC, SSA-BIC, and the chi-square difference tests using the loglikelihood and scaling correction factors (Muthén & Muthén, 2017).

##### Links of Cognitive Emotion Regulation Profiles with Internalizing Problems

We examined the cross-sectional associations of profile membership with internalizing problems at each timepoint, controlling for child gender and the type of school youths attended (i.e., regular or special education). This resulted in the estimation of one path model with three dependent variables (i.e., internalizing problems at Wave 1, Wave 2, and Wave 3), each of them predicted by *k*−1 independent variables (at Wave 1, Wave 2, and Wave 3, respectively), with *k* being the number of profiles selected in the previous analyses. 

The missing data and interdependency analyses were conducted in R Studio (RStudio Team, 2020). The CFAs and the main analyses were conducted in Mplus, version 8 (Muthén & Muthén, 2017). Full Information Maximum Likelihood (FIML) was used to handle missingness in the CFAs, the LPAs, and the LPTA. We used a cutoff of *p* < .05 to test the significance of predictive effects. The data/materials/code are not openly available. They are available upon request. This study’s design and its analysis were not pre-registered.

## Results

### Descriptive Statistics

Descriptive statistics and correlations between the study variables are provided in Tables S1 and S2. Means of the cognitive emotion regulation strategies were generally slightly below the midpoint of the scale and most maladaptive strategies (e.g., blaming others at Wave 1: *M* = 1.91) were descriptively somewhat lower than the adaptive strategies (e.g., positive refocusing at Wave 1: *M* = 3.1). The use of cognitive emotion regulation strategies tended to be intercorrelated within waves, with most strategies showing positive associations indicating that youths who tended to use one strategy tended to also use others. Descriptive statistics generally show a pattern similar to that in previous research using the 36-item CERQ-k (Garnefski et al., 2007). In general, more maladaptive strategies (e.g., catastrophizing) were positively associated with internalizing problems, whereas adaptive strategies (e.g., positive reappraisal) were negatively associated with internalizing problems.

### Clustering of the Cognitive Emotion Regulation Strategies

Inspecting the statistical criteria outlined in the Method did not consistently point to one solution as being most appropriate for our data (Table 1). When carefully considering the profile solutions across waves, we saw that the three- and four-profile solutions were most consistently showing the best fit statistics, clarity, and accuracy, as well as large enough sample sizes. We evaluated the content of the three- and four-profile solutions in terms of their conceptual meaningfulness. The three-profile solution yielded meaningful profiles that were similar across waves; the four-profile solution revealed the same profiles as the three-profile solution for all waves, including a fourth profile that was conceptually less clearly distinguishable across waves. Therefore, we determined that a three-profile LPA solution was the optimal, most parsimonious solution for all three waves. The largest profile (55.5% at Wave 1, 56.9% at Wave 2, and 51.7% at Wave 3) was characterized by scores below the midpoint of the scale for all 9 cognitive emotion regulation strategies, with particularly lower scores on the more maladaptive strategies. The second profile (31.1%, 33.0%, and 27.8% at Wave 1–3, respectively) was characterized by relatively high scores on the adaptive strategies and lower scores on the maladaptive strategies. The last profile (13.4%, 10.1%, and 20.4% at Wave 1–3, respectively) was characterized by below-average scores on the adaptive strategies and generally higher scores on the maladaptive strategies. Regarding child gender, girls were more likely to be in the third profile than in the second profile at Wave 1 (odds ratio: 2.52), more likely to be in the third profile than in the first profile and the second profile at Wave 2 (odds ratio: 11.09, CI_95%_ = [1.95;63.04] and 6.38, CI_95%_ = [1.07;38.13], respectively), and more likely to be in the third profile than in the first profile at Wave 3 (odds ratio: 2.27, CI_95%_ = [1.04;4.95]).

**Table 1:** LPA fit

						Percentage in each profile				
	BIC	SSA-BIC	VLMR-LRT *p*	Entropy	ACPMLC	1	2	3	4	5
*Wave 1*										
1	12905.08	12847.95				1.00				
2	12581.50	12492.63	< 0.001	0.72	0.93	0.63	0.37			
3	12435.96	12315.35	0.164	0.79	0.92	0.57	0.13	0.31		
4	12320.49	12168.14	0.250	0.80	0.90	0.10	0.26	0.11	0.53	
5	12280.89	12096.80	0.023	0.84	0.92	0.50	0.06	0.17	0.24	0.02
*Wave 2*										
1	4287.20	4230.19				1.00				
2	4220.92	4132.24	0.179	0.71	0.93	0.61	0.39			
3	4155.22	4034.89	0.087	0.81	0.97	0.09	0.56	0.34		
4	4163.62	4011.62	0.126	0.84	0.93	0.54	0.10	0.07	0.29	
5	4175.60	3991.92	0.234	0.83	0.86	0.30	0.07	0.46	0.06	0.11
*Wave 3*										
1	5580.67	5523.63				1.00				
2	5439.61	5350.88	< 0.001	0.93	0.99	0.79	0.21			
3	5376.08	5255.67	0.202	0.81	0.93	0.53	0.20	0.27		
4	5353.41	5201.31	0.293	0.86	0.93	0.55	0.21	0.15	0.08	
5	5352.43	5168.65	0.158	0.88	0.94	0.57	0.09	0.18	0.08	0.08

**Fig. 1:**
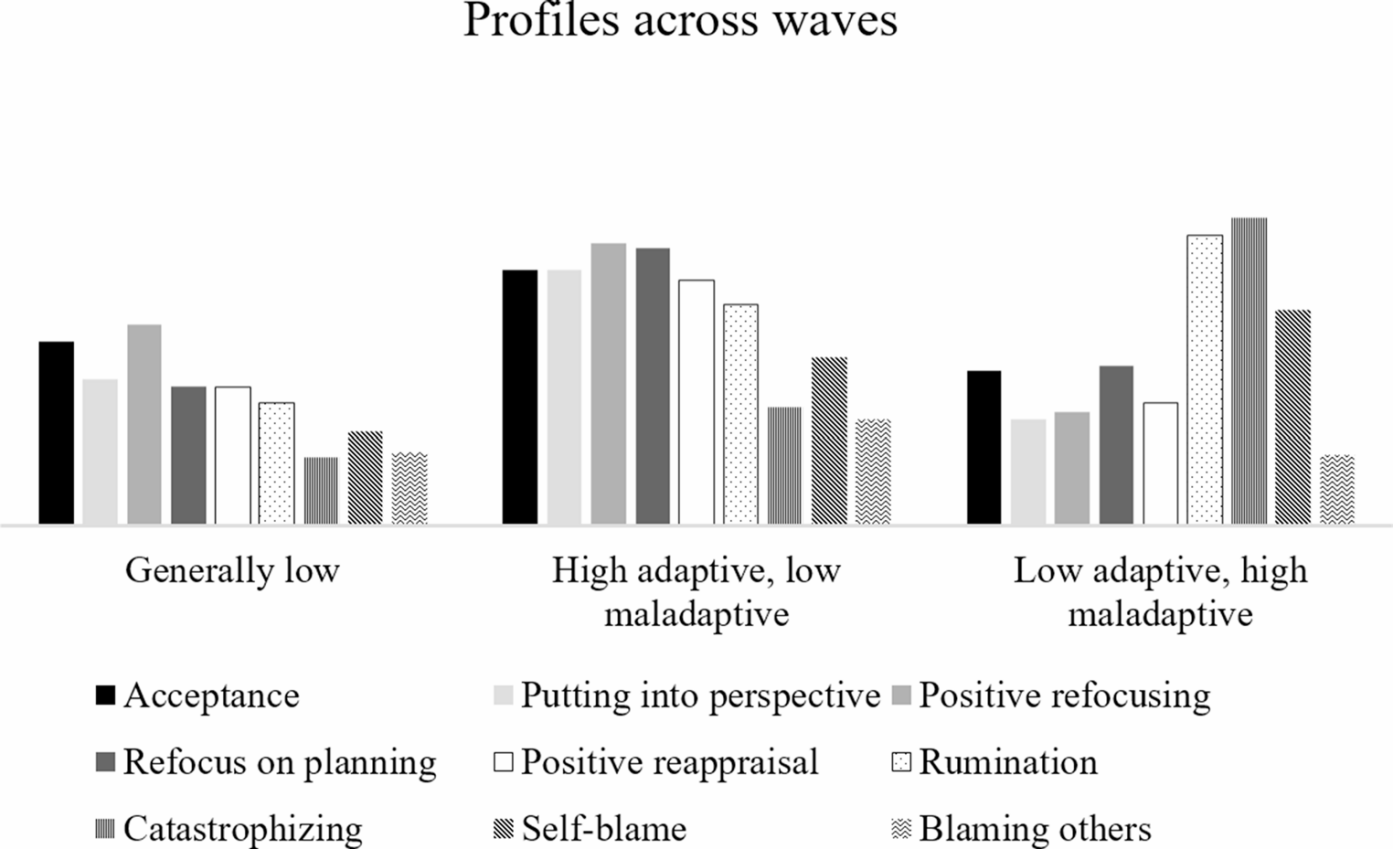
Visualization of the three LPTA profiles

Next, we fit an LPTA with three profiles at each wave. In addition, to test whether this solution was the best-fitting solution, we also fit models with two profiles, with four profiles, and a 3-profile solution that allowed means in profiles to vary over time. Comparing these different solutions, we determined that although all χ^2^ tests were significant, the decrease in BIC and SSA-BIC was decidedly larger from the 2-profile to the 3-profile solution (around 400) than from the 3-profile to the 4-profile solution (around 200; Table 2). Moreover, the non-invariant 3-profile solution had a higher BIC and SSA-BIC than the invariant solution. Therefore, the 3-profile, fully invariant solution was determined to be most appropriate for the data. The same general patterns emerged as in the LPA, with a profile with generally low scores on all cognitive emotion regulation strategies (49.2%, 52.3%, and 52.3% at Wave 1–3, respectively), a profile with relatively high scores on the adaptive strategies and lower scores on the maladaptive strategies (36.3%, 34.2%, and 30.9%, respectively), and a profile with below-average scores on the adaptive strategies and relatively high scores on the maladaptive strategies (14.5%, 13.5%, and 16.9%, respectively). More information on the profiles can be found in Table 3 (visual representation in Fig. 1).^4^

**Table 2:** LPTA fit

Number of profiles	Invariance	BIC	SSA-BIC	LL	SCF	χ^2^ (df)	*p*
2	Full	22067.10	21908.40	−10878.08	1.24		
3	Full	21640.31	21424.47	−10608.72	1.26	409.50 (18)	< 0.001
4	Full	21481.45	21195.78	−10460.89	1.35	181.59 (22)	< 0.001
3	Non-invariant	21869.15	21481.91	−10555.24	1.17	101.22 (54)	< 0.001

*N* = 502

**Table 3:** Description of the profiles of the LPTA

	Proportion						Unstandardized mean score					
	Wave 1	Wave 2	Wave 3	Acceptance	Putting into perspective	Positive refocusing	Refocus on planning	Positive reappraisal	Rumination	Catastro-phizing	Self-blame	Blaming others
Generally low	49.2	52.3	52.3	2.73	2.38	2.88	2.31	2.30	2.15	1.64	1.89	1.69
High adaptive, low maladaptive	36.3	34.2	30.9	3.40	3.40	3.65	3.60	3.30	3.07	2.12	2.57	2.03
Low adaptive, high maladaptive	14.5	13.5	16.9	2.45	2.00	2.07	2.50	2.15	3.72	3.89	3.02	1.67

*N* = 502

We found 13 different transition patterns across the three waves of the total 27 possible patterns (3 profiles and 3 waves). Several of these patterns only occurred a few times. We only interpreted patterns that occurred enough as to be robust, as determined by the confidence interval not containing zero. This resulted in a total of ten interpretable patterns, across 493 participants (see Table 4). Across the board, we saw most evidence of stability, with 79.5% of the sample remaining in the same status across all three waves. Most youths remained stable in the first profile (45.2%) and less participants were stable in the second, most adaptive profile (24.3%) and the least number of participants remained in the third, least adaptive profile (9.9%). Of the participants who transitioned between waves, none did so more than once. Moreover, most of the transitions occurred from the first to second measurement occasion. For youths transitioning to another profile, the first profile was the most common endpoint (53.5%), followed by the second profile (32.7%) and then the third profile (13.9%). Moreover, of youths who transitioned, most transitioned towards a less adaptive endpoint (61.4%; 12.4% of the total sample) and fewer towards a more adaptive endpoint (38.6%; 7.9% of the total sample).

**Table 4:** LPTA patterns

Wave 1	Wave 2	Wave 3	Frequency	95% CI frequency	Percentage	95% CI Percentage
Generally low	Generally low	Generally low	223	202; 244	45.23	40.88; 49.59
High adaptive, low maladaptive	High adaptive, low maladaptive	High adaptive, low maladaptive	120	101; 139	24.34	20.59; 28.09
Low adaptive, high maladaptive	Low adaptive, high maladaptive	Low adaptive, high maladaptive	49	36; 62	9.94	7.32; 12.56
High adaptive, low maladaptive	Generally low	Generally low	41	29; 53	8.32	5.90; 10.73
Generally low	High adaptive, low maladaptive	High adaptive, low maladaptive	23	14; 32	4.67	2.82; 6.51
Low adaptive, high maladaptive	High adaptive, low maladaptive	High adaptive, low maladaptive	10	4; 16	2.03	0.80; 3.26
High adaptive, low maladaptive	Low adaptive, high maladaptive	Low adaptive, high maladaptive	8	3; 13	1.62	0.52; 2.73
High adaptive, low maladaptive	High adaptive, low maladaptive	Generally low	7	2; 12	1.42	0.38; 2.45
Generally low	Low adaptive, high maladaptive	Low adaptive, high maladaptive	6	1; 11	1.22	0.26; 2.18
Low adaptive, high maladaptive	Generally low	Generally low	6	1; 11	1.22	0.26; 2.18

*N* = 493

### Links of Cognitive Emotion Regulation Profiles with Internalizing Problems

We examined the cross-sectional associations of the three cognitive emotion regulation profiles with internalizing problems, controlling for child gender and type of school.^5^ We made use of dummy coding for profile membership, so that the first profile (i.e., “Generally low”) was the reference group and the second and third profiles (i.e., “High adaptive, low maladaptive” and “Low adaptive, high maladaptive”, respectively) were the dummies. Youths who were in the third profile (who relied relatively much on maladaptive cognitive emotion regulation strategies) had more parent-reported internalizing problems as compared to youths who made relatively little use of all strategies, at Wave 1 and 2, but not at Wave 3 (Table 5). Youths making relatively much use of adaptive cognitive emotion regulation strategies did not differ significantly from those in profile 1 at Wave 1 and 3, but did have more parent-reported internalizing problems at Wave 2.

**Table 5:** Cross-sectional associations between cognitive emotion regulation strategy profiles and internalizing problems at each timepoint

	b	β	*p*
Wave 1			
Gender (girl)	0.01	0.02	0.771
Type of school (special education)	0.53	0.52	< 0.001
High adaptive, low maladaptive	−0.004	−0.01	0.843
Low adaptive, high maladaptive	0.13	0.18	0.001
Wave 2			
Gender (girl)	−0.03	−0.08	0.332
Type of school (special education)	0.32	0.43	< 0.001
High adaptive, low maladaptive	0.05	0.14	0.008
Low adaptive, high maladaptive	0.08	0.14	0.016
Wave 3			
Gender (girl)	0.02	0.06	0.460
Type of school (special education)	0.30	0.41	< 0.001
High adaptive, low maladaptive	0.01	0.04	0.514
Low adaptive, high maladaptive	−0.003	−0.01	0.920

*N* = 502. Reference group was “Generally low” profile (profile 1). For gender, boys were the reference group. For type of school, regular education was the reference group

## Discussion

The aim of the current study was to extend previous research by examining profiles of cognitive emotion regulation strategy use not only cross-sectionally but also longitudinally, in the developmental transition from childhood to adolescence (ages 10 to 12). First, we cross-sectionally identified three cognitive emotion regulation profiles. Consistent with our hypothesis, we identified a “Generally low” profile, indicating generally little use of all strategies. This profile was identified in most youths at all ages (55.5%, 56.9%, and 51.7% of the youths at Wave 1–3, respectively). Contrary to our hypothesis, we did not identify a profile indicating much use of all strategies. As hypothesized, we identified two profiles with specific combinations of cognitive emotion regulation strategies: a “High adaptive, low maladaptive” profile (31.1%, 33.0%, and 27.8% at Wave 1–3, respectively), and a “Low adaptive, high maladaptive” profile (13.4%, 10.1%, and 20.4% at Wave 1–3, respectively). Thus, the composition of the emotion regulation repertoire was consistent with the composition found in most other studies on older adolescents and adults (Chesney & Gordon, 2017; Chesney et al., 2019; Dixon-Gordon et al., 2015).

Second, we found longitudinal evidence for stability and change in cognitive emotion regulation profiles. Consistent with our hypothesis, most youths (79.5% of the sample) remained stable in their cognitive emotion regulation profiles across the three waves. As hypothesized—though we based our hypotheses on cross-sectional person-centered studies and variable-centered studies due to the lack of longitudinal person-centered studies—transitions occurred. However, we found no evidence for the expected transitions towards more frequent use of all cognitive emotion regulation strategies, as we did not identify a profile consisting of much use of all strategies in all waves. Transitions towards a less adaptive profile were most common (61.4%; 12.4% of the total sample), followed by transitions towards a more adaptive profile (38.6%; 7.9% of the total sample). Overall, the findings align with theories conceptualizing the habitual use of emotion regulation strategies as fairly stable trait-like characteristics (Gross & John, 2003; Liu & Thompson, 2017).

The absence of a profile indicating much use of all cognitive emotion regulation strategies may be explained by the age of our participants. Youths aged 10 to 12 may not yet feel the need to regulate emotions extensively (Gross et al., 2019), which could explain why more than half of our sample made little use of strategies. Previous studies suggest that younger adolescents make less use of strategies than older adolescents (Chesney & Gordon, 2017; Garnefski & Kraaij, 2006b; van den Heuvel et al., 2020). It may take time for youths to adapt to increases in emotional reactivity, that typically occur after puberty starts (ages 9 − 12; girls usually 1 or 2 years earlier than boys; Casey et al., 2010; Crone & Dahl, 2012), and to make use of emotion regulation strategies. Cognitive strategies may not yet be the dominant emotion regulation strategies in this developmental period; other strategies, such as behavioral strategies (e.g., distraction, which is employed more by children than by adolescents; Cracco et al., 2017), may be employed more frequently. Youths’ cognitive skills are still developing, which could make it difficult to effectively use cognitive emotion regulation strategies (Casey et al., 2010; Ochsner & Gross, 2008; Somerville et al., 2010). However, explanations are purely speculative, as no studies have examined cognitive emotion regulation profiles in this age group.

Our finding of high stability in profiles does not imply that there is no development. In the personality literature, stable adaptive profiles and transitions towards more adaptive profiles have been described as indicators of maturation (Klimstra et al., 2009, 2012; Laceulle et al., 2023; Roberts et al., 2001). However, a stable maladaptive emotion regulation profile and transitions towards a less adaptive emotion regulation profile may indicate stagnation in maturation, possibly related to pubertal changes. Our finding that most adolescents remain stable in their profiles may also be explained by the short time interval between our measures. Change in cognitive emotion regulation strategy use may take place over a longer period of time or later in adolescence. Previous studies that examined wider age ranges suggest that developmental changes occur across childhood, adolescence, and young adulthood, though most studies focused on older adolescents (e.g., Cracco et al., 2017; van den Heuvel et al., 2020; Zimmermann & Iwanski, 2014). In our study, transitions occurred mainly between ages 10 and 11, around puberty’s onset (Crone & Dahl, 2012). Switching back and forth between profiles multiple times did not occur. Future research should explore risk and protective factors associated with maladaptive stability and transitions in cognitive emotion regulation profiles.

We additionally examined the adaptive or maladaptive nature of the profiles by testing cross-sectional associations with parent-reported internalizing problems. The maladaptive nature of the “Low adaptive, high maladaptive” profile was confirmed cross-sectionally at Wave 1 and 2. The adaptive nature of the “High adaptive, low maladaptive” profile was not confirmed, contrary to our hypothesis. Youths in this profile did not differ from the youths who made little use of all strategies in parent-reported internalizing problems at waves 1 and 3, and had even more parent-reported internalizing problems at wave 2, possibly because it is not age-appropriate to already use a lot of cognitive strategies. Emotion regulation strategies other than cognitive ones—such as behavioral strategies—might play a more significant role in this age group. The findings suggest that a profile characterized by below-average use of adaptive strategies and much use of maladaptive strategies is particularly maladaptive. However, it can be debated what such a maladaptive profile reflects as using a different conceptualization of emotion regulation may yield different profiles.

Associations between the “Low adaptive, high maladaptive” cognitive emotion regulation profile and internalizing problems were only confirmed at two waves and we have no information on longitudinal associations, because of which caution is required in drawing strong conclusions. The assessment of cognitive emotion regulation and internalizing problems may also influence study findings. We asked children to report on their use of cognitive emotion regulation strategies and parents to report on their child’s internalizing problems, thereby reducing the risk of shared method bias across measures. However, we do not know whether cognitive emotion regulation profiles were associated with the internal aspects of internalizing problems experienced by the youths or only the more outward symptoms visible to parents. Moreover, not all children may have the cognitive skills to accurately reflect on their cognitive-emotional responses to negative events (Casey et al., 2010). Combining parent and child perspectives in the assessment of both cognitive emotion regulation and internalizing problems may offer a more comprehensive view of these constructs (Martel et al., 2017).

Although we did not identify a profile reflecting an extensive repertoire, our results did not support the suggestion that a more extensive repertoire is more adaptive than a narrow repertoire (Aldao et al., 2015; Bonanno & Burton, 2013), as we did not find that the “High adaptive, low maladaptive” profile was more adaptive than the narrow “Generally low” profile. The adaptiveness of the size of the emotion regulation repertoire might be more evident in other outcomes, such as immediate emotional experiences in specific contexts. Studies using Ecological Momentary Assessment (EMA) may shed more light on when cognitive emotion regulation strategies are adaptive or maladaptive in the moment, considering the contextual demands (Aldao et al., 2015; Bonanno & Burton, 2013). Furthermore, we do not know the direction of effects, because internalizing problems may also cause youths to have to deal with a lot of stressors that cause them to use more maladaptive cognitive emotion regulation strategies. Further research is needed to learn more about longitudinal associations between cognitive emotion regulation profiles and changes in internalizing problems over longer periods in time.

Findings from this study suggest that the transition from childhood to adolescence may be a suitable period for interventions targeting cognitive emotion regulation because there appears to be some flexibility, as profile transitions occur. Difficulties in cognitive emotion regulation may also begin to emerge during this developmental period. As prefrontal regions mature and cognitive abilities develop (Casey et al., 2010; Ochsner & Gross, 2008; Somerville et al., 2010), early adolescents likely have the cognitive abilities to benefit from interventions targeting emotion regulation skills, also shown in previous research (Moltrecht et al., 2021; Volkaert et al., 2022). Younger children may not yet have the cognitive abilities to implement cognitive emotion regulation strategies, such as positive reappraisal (Cracco et al., 2017). Interventions could be tailored to youths’ cognitive emotion regulation repertoire to increase their benefit from the intervention.

Several limitations should be noted. First, our sample was limited in that it was a convenience sample and a relatively small sample to test our research questions. We recruited youths at 24 primary schools in the Netherlands, including schools for special education. However, we did not recruit in all parts of the Netherlands and there was longitudinal dropout, because of which our sample was not fully representative of the Netherlands. A larger and more clinically diverse sample may have allowed the testing of longitudinal associations between transitions in cognitive emotion regulation profiles and change in internalizing problems. Second, each cognitive emotion regulation dimension was assessed by two items only. While most reliability statistics were acceptable, for some strategies internal consistency was relatively low at all waves (i.e., positive reappraisal, refocus on planning, and acceptance). As such our findings should be interpreted with caution. The results of this study should be replicated using the 36-item CERQ-kids version (Garnefski et al., 2007). Third, this study focused on the habitual use of cognitive emotion regulation strategies across situations and, as noted earlier, did not consider the context. Emotion polyregulation, that is, the use of multiple emotion regulation strategies within a real-life emotional episode (Ford et al., 2019), can also not be studied with our approach; this requires EMA.

The findings of this study on the transitions between cognitive emotion regulation profiles should be replicated in future research, preferably using larger samples and examining longer time periods from childhood to adolescence and later in life. To map the developmental implications of cognitive emotion regulation profile transitions, associations with specific trajectories of internalizing problems could be examined. To fully understand emotion regulation profiles, behavioral strategies should be included. The current study is a first attempt to examine cognitive emotion regulation profile transitions and demonstrates the need to further clarify patterns of emotion regulation within individuals over time.

## Conclusion

To summarize, this person-centered study cross-sectionally identified three cognitive emotion regulation profiles at ages 10, 11, and 12 years: (1) “Generally low” profile, (2) “High adaptive, low maladaptive” profile, and (3) “Low adaptive, high maladaptive” profile. Across the three waves, most youths remained stable in their cognitive emotion regulation profiles but transitions also occurred: most towards a less adaptive profile and fewer towards a more adaptive profile. The maladaptive nature of the “Low adaptive, high maladaptive” profile was confirmed by cross-sectional associations with parent-reported internalizing problems at Wave 1 and 2 but not Wave 3. The findings suggest that a more maladaptive combination of cognitive strategies has more clinical relevance than the number of cognitive strategies youths use during the transitional period from childhood to adolescence, though this is only based on cross-sectional associations with parent-reported internalizing problems. This study shows for the first time stability in cognitive emotion regulation profiles, but also that transitions occur. The transition from childhood to adolescence may be an important age period for intervention, as there is some developmental change in youths’ cognitive emotion regulation profiles.

## Supplementary Information

Below is the link to the electronic supplementary material.

Supplementary Material 1 (DOCX 83.9 KB)

## Data Availability

The data and materials are not openly available. They are available upon request.

## Footnotes

1 For clarity, in line with this research on the habitual use of cognitive emotion regulation strategies we refer to specific strategies as adaptive or maladaptive, acknowledging that a strategy can be adaptive or maladaptive depending on the context (Aldao et al., 2015; Bonanno & Burton, 2013).

2 Initially, we aimed to examine longitudinal associations between cognitive emotion regulation profiles and change in internalizing problems to explore whether a transition towards a less adaptive profile is associated with an increase in internalizing problems. However, due to the poor model fit of the tested model, we do not report any findings on this in the main text.

3 Item was excluded from reliability analysis because it caused issues with estimation. However, the item was still included in the other analyses.

4 We checked the robustness of our LPA and LPTA findings by rerunning the analyses on two random halves of the dataset. As can be seen in Tables S3-S5 and Figure S1 and Tables S6-S8 and Figure S2, respectively, the findings were very similar across the two halves and to the results based on the full dataset.

5 In addition, we reran these cross-sectional analyses on the subsample of youths who were in regular education, to check whether our findings were not driven solely by the group in special education given the strong design effects of type of school on internalizing problems. The additional analyses (Table S9) revealed no meaningful differences from the main analyses, suggesting that the associations were not merely driven by the subgroup of participants with more internalizing problems.
