# Supplementary material for: Developmental Change in Cognitive Emotion Regulation Profiles in the Transition from Childhood to Adolescence
Source: Res Child Adolesc Psychopathol. 2025 Oct 7;53(12):2003–16. doi: 10.1007/s10802-025-01375-1 (PMC12718262; doi:10.1007/s10802-025-01375-1)
Supplement: Supplementary file 1 — Supplementary Material 1 (DOCX 83.9 KB) [file 10802_2025_1375_MOESM1_ESM.docx]

Supplemental Materials

**Missingness and dropout**

We examined whether child gender, parental gender, and parental education were related to missingness in the dataset, and whether these background variables and baseline scores of the use of cognitive emotion regulation strategies and of the level of internalizing problems were related to dropout across waves. Across waves, there was on average 27.8% missing data in the cognitive emotion regulation and internalizing problems variables. Participants who identified as boys or girls or whose parents identified as man or woman did not significantly differ in their percentage of missing data (*t*(495) = -1.95, *p* = .052 and *t*(382) = 0.03, *p* = .973, respectively).

Furthermore, there was substantial dropout across waves, with 59.7% of participants still participating at Wave 2 and 42.2% at Wave 3. Comparing participants who remained in the study with those who dropped out, we found that the two groups did not differ significantly in the distribution of the gender of the participant (χ^2^(1) < .01, *p* > .999) or their parent (χ^2^(1) = 1.60, *p* = .207), nor on parental education level (*t*(224) = 1.84, *p* = .068). The two groups also did not differ at Wave 1 on their mean scores on acceptance, putting into perspective, positive refocusing, refocus on planning, positive reappraisal, rumination, catastrophizing, self-blame, and blaming others (*t*(482) = 0.48, *p* .634, *t*(484) = 0.43, *p* = .664, *t*(480) = -0.94, *p* = .350, *t*9482) = -0.419, *p* = .675, *t*(483) = -0.35, *p* = .723, *t*(483) = -0.76, *p* = .449, *t*(484) = -0.73, *p* = .464, *t*(482) = 1.61, *p* = .108, *t*(477.76), = -1.09, *p* = .274, respectively), nor on internalizing problems (*t*(350.27) = 1.55, *p* = .123).

**Structure of the CBCL internalizing problems scale**

We checked the structure of the CBCL internalizing problems scale. We estimated a one-factor CFA based on the higher-order factor for internalizing problems identified by Achenbach and Rescorla (2001). Because of the large number of items, we randomly assigned items to parcels to decrease model complexity (Little et al., 2013). In particular, we created ten parcels, of which seven contained 3 items, and three contained 4 items. The ten parcels were included as indicators of the latent internalizing problems factor. The model fit in the three waves was acceptable (χ^2^(31) = 67.25, *p* < .001, CFI = .982, RMSEA = .056, χ^2^(31) = 73.87, *p* < .001, CFI = .973, RMSEA = .076, χ^2^(31) = 48.38, *p* = .024, CFI = .973, RMSEA = .056, respectively).

To examine whether the structure of the CBCL internalizing problems scale was invariant across time, we fit a series of increasingly constrained models. Specifically, we fitted a model with the same model structure including covariances across time (configural invariance; χ^2^(360) = 786.54, *p* < .001, CFI = .922, RMSEA = .053), a model with additionally constrained factor loadings (weak invariance; χ^2^(378) = 827.20, *p* < .001, CFI = .918, RMSEA = .053), additionally constrained item intercepts (strong invariance; χ^2^(398) = 876.70, *p* < .001, CFI = .912, RMSEA = .054), and additionally constrained residual variances (strict invariance; χ^2^(418) = 937.69, *p* < .001, CFI = .905, RMSEA = .054). We compared these models based on changes in the CFI and the RMSEA model fit indices, where changes of ≥ -0.01 in the CFI in addition to changes of ≥ 0.015 in the RMSEA were seen as indicative of a significant change in model fit and thus non-invariance (Chen, 2007). We concluded that strict invariance held for the CBCL internalizing problems data from Wave 1 to Wave 3.

**Table S1**

*Descriptive statistics of the study variables*

|  |  | |  |  |  |  |  |  |  |  |  |  |  |  |  |  |
| --- | --- | --- | --- | --- | --- | --- | --- | --- | --- | --- | --- | --- | --- | --- | --- | --- |
|  | Wave 1 | | | | | | Wave 2 | | | | | Wave 3 | | | | |
|  | *n* | | Mean | *SD* | min | max | *n* | Mean | *SD* | min | max | *n* | Mean | *SD* | min | max |
| Acceptance | | 484 | 2.84 | 1.03 | 1 | 5 | 177 | 3.02 | 1 | 1 | 5 | 213 | 3.02 | 1.04 | 1 | 5 |
| Putting into perspective | | 486 | 2.69 | 1.03 | 1 | 5 | 177 | 2.73 | 0.91 | 1 | 5 | 213 | 2.56 | 1.1 | 1 | 5 |
| Positive refocusing | | 482 | 3.08 | 1.16 | 1 | 5 | 176 | 2.94 | 0.99 | 1 | 5 | 212 | 2.98 | 1.09 | 1 | 5 |
| Refocus on planning | | 484 | 2.83 | 1.03 | 1 | 5 | 177 | 2.78 | 0.95 | 1 | 5 | 213 | 2.67 | 1.01 | 1 | 5 |
| Positive reappraisal | | 485 | 2.66 | 1.02 | 1 | 5 | 177 | 2.58 | 0.85 | 1 | 5 | 213 | 2.57 | 0.91 | 1 | 5 |
| Rumination | | 485 | 2.78 | 1.07 | 1 | 5 | 177 | 2.64 | 0.97 | 1 | 5 | 213 | 2.54 | 1.08 | 1 | 5 |
| Catastrophizing | | 486 | 2.26 | 1.18 | 1 | 5 | 177 | 1.97 | 0.95 | 1 | 5 | 213 | 2.08 | 1.17 | 1 | 5 |
| Self-blame | | 484 | 2.31 | 1.03 | 1 | 5 | 177 | 2.27 | 0.9 | 1 | 5 | 213 | 2.25 | 1.02 | 1 | 5 |
| Blaming others | | 486 | 1.91 | 0.93 | 1 | 5 | 177 | 1.75 | 0.68 | 1 | 4 | 213 | 1.68 | 0.77 | 1 | 5 |
| Internalizing problems | | 370 | 1.18 | 0.21 | 1 | 2.33 | 242 | 1.17 | 0.21 | 1 | 3 | 176 | 1.16 | 0.17 | 1 | 1.82 |

**Table S2**

*Correlations between the study variables*

|  | 1. | 2. | 3. | 4. | 5. | 6. | 7. | 8. | 9. | 10. | 11. | 12. |
| --- | --- | --- | --- | --- | --- | --- | --- | --- | --- | --- | --- | --- |
| 1. Acceptance W1 |  |  |  |  |  |  |  |  |  |  |  |  |
| 2. Putting into perspective W1 | 0.28* |  |  |  |  |  |  |  |  |  |  |  |
| 3. Positive refocusing W1 | 0.26* | 0.30* |  |  |  |  |  |  |  |  |  |  |
| 4. Refocus on planning W1 | 0.22* | 0.44* | 0.33* |  |  |  |  |  |  |  |  |  |
| 5. Positive reappraisal W1 | 0.25* | 0.38* | 0.33* | 0.47* |  |  |  |  |  |  |  |  |
| 6. Rumination W1 | 0.16* | 0.13* | -0.02 | 0.29* | 0.22* |  |  |  |  |  |  |  |
| 7. Catastrophizing W1 | 0.00 | -0.03 | -0.15* | 0.04 | -0.03 | 0.54* |  |  |  |  |  |  |
| 8. Self-blame W1 | 0.21* | 0.13* | -0.05 | 0.19* | 0.15* | 0.28* | 0.34* |  |  |  |  |  |
| 9. Blaming others W1 | 0.11* | 0.18* | 0.04 | 0.16* | 0.12* | 0.16* | 0.23* | 0.08 |  |  |  |  |
| 10. Acceptance W2 | 0.16* | 0.11 | -0.03 | 0.04 | 0.04 | -0.06 | -0.16* | -0.10 | -0.11 |  |  |  |
| 11. Putting into perspective W2 | 0.09 | 0.15* | 0.17* | 0.26* | 0.10 | -0.02 | -0.15 | -0.02 | 0.06 | 0.25* |  |  |
| 12. Positive refocusing W2 | 0.14 | 0.17* | 0.34* | 0.17* | 0.09 | -0.08 | -0.20* | -0.12 | -0.03 | 0.29* | 0.36* |  |
| 13. Refocus on planning W2 | 0.24* | 0.1 | 0.17* | 0.33* | 0.16* | 0.12 | -0.06 | 0.03 | 0.03 | 0.16* | 0.43* | 0.21* |
| 14. Positive reappraisal W2 | 0.06 | 0.08 | 0.13 | 0.17* | 0.14 | 0.02 | -0.19* | -0.04 | -0.01 | 0.21* | 0.32* | 0.17* |
| 15. Rumination W2 | 0.04 | 0.06 | -0.04 | 0.06 | 0.09 | 0.36* | 0.22* | 0.12 | -0.04 | 0.05 | 0.03 | -0.08 |
| 16. Catastrophizing W2 | -0.02 | 0.10 | -0.11 | -0.00 | -0.03 | 0.33* | 0.39* | 0.09 | -0.01 | -0.16* | -0.17* | -0.23* |
| 17. Self-blame W2 | 0.07 | -0.09 | -0.10 | -0.11 | -0.01 | 0.10 | 0.13 | 0.32* | 0.07 | -0.04 | 0.10 | -0.12 |
| 18. Blaming others W2 | -0.08 | 0.05 | -0.01 | 0.12 | 0.05 | 0.01 | -0.11 | -0.18* | 0.28* | 0.06 | 0.26* | 0.12 |
| 19. Acceptance W3 | 0.10 | -0.05 | 0.04 | -0.06 | 0.01 | -0.11 | -0.20* | -0.09 | -0.04 | 0.75* | 0.28* | 0.19* |
| 20. Putting into perspective W3 | 0.08 | 0.13 | 0.06 | 0.22* | 0.10 | -0.00 | -0.03 | 0.07 | 0.06 | 0.22* | 0.74* | 0.26* |
| 21. Positive refocusing W3 | 0.13 | 0.02 | 0.23* | 0.14 | 0.09 | -0.06 | -0.11 | -0.11 | 0.04 | 0.18* | 0.34* | 0.66* |
| 22. Refocus on planning W3 | 0.25* | 0.06 | 0.20* | 0.35* | 0.20* | 0.13 | -0.03 | 0.12 | 0.05 | 0.10 | 0.42* | 0.12 |
| 23. Positive reappraisal W3 | 0.11 | 0.08 | 0.11 | 0.28* | 0.27* | 0.11 | -0.03 | 0.08 | 0.02 | 0.32* | 0.26* | 0.25* |
| 24. Rumination W3 | 0.06 | 0.00 | -0.03 | 0.01 | 0.04 | 0.27* | 0.18* | 0.18* | -0.04 | 0.04 | 0.06 | -0.05 |
| 25. Catastrophizing W3 | -0.02 | 0.05 | -0.07 | -0.07 | -0.06 | 0.17* | 0.27* | 0.14* | 0.02 | -0.19* | -0.15 | -0.22* |
| 26. Self-blame W3 | 0.07 | -0.01 | -0.06 | -0.08 | 0.01 | 0.12 | 0.20* | 0.27* | 0.05 | 0.03 | 0.06 | -0.12 |
| 27. Blaming others W3 | -0.07 | 0.00 | 0.03 | 0.15* | 0.06 | -0.07 | -0.09 | -0.05 | 0.33* | 0.09 | 0.32* | 0.14 |
| 28. Internalizing problems W1 | -0.04 | -0.10 | -0.17* | -0.15* | -0.16* | 0.11* | 0.24* | 0.04 | 0.12* | -0.23* | -0.11 | -0.18* |
| 29. Internalizing problems W2 | -0.04 | -0.20* | -0.08 | -0.12 | -0.16* | 0.11 | 0.26* | 0.05 | 0.19* | -0.22* | -0.06 | -0.12 |
| 30. Internalizing problems W3 | -0.03 | -0.12 | -0.07 | -0.07 | -0.09 | 0.05 | 0.11 | 0.00 | 0.15 | -0.21* | -0.10 | -0.18* |
|  | 13. | 14. | 15. | 16. | 17. | 18. | 19. | 20. | 21. | 22. | 23. | 24. |
| 14. Positive reappraisal W2 | 0.35* |  |  |  |  |  |  |  |  |  |  |  |
| 15. Rumination W2 | 0.23* | 0.19* |  |  |  |  |  |  |  |  |  |  |
| 16. Catastrophizing W2 | 0.05 | -0.11 | 0.50* |  |  |  |  |  |  |  |  |  |
| 17. Self-blame W2 | 0.39* | 0.17* | 0.29* | 0.31* |  |  |  |  |  |  |  |  |
| 18. Blaming others W2 | 0.04 | 0.14 | -0.01 | 0.03 | -0.15* |  |  |  |  |  |  |  |
| 19. Acceptance W3 | 0.17* | 0.22* | -0.01 | -0.24* | 0.03 | -0.00 |  |  |  |  |  |  |
| 20. Putting into perspective W3 | 0.36* | 0.23* | 0.00 | -0.16* | 0.10 | 0.12 | 0.25* |  |  |  |  |  |
| 21. Positive refocusing W3 | 0.25* | 0.26* | -0.04 | -0.28* | -0.15 | 0.13 | 0.18* | 0.35* |  |  |  |  |
| 22. Refocus on planning W3 | 0.74* | 0.27* | 0.15* | 0.01 | 0.32* | 0.06 | 0.10 | 0.38* | 0.25* |  |  |  |
| 23. Positive reappraisal W3 | 0.34* | 0.64* | 0.09 | -0.12 | 0.01 | 0.12 | 0.27* | 0.27* | 0.37* | 0.39* |  |  |
| 24. Rumination W3 | 0.28* | 0.17* | 0.81* | 0.44* | 0.33* | -0.04 | 0.02 | 0.01 | -0.05 | 0.21* | 0.09 |  |
| 25. Catastrophizing W3 | -0.01 | -0.12 | 0.41* | 0.81* | 0.32* | 0.02 | -0.17* | -0.14* | -0.33* | -0.02 | -0.15* | 0.51* |
| 26. Self-blame W3 | 0.21* | 0.06 | 0.18* | 0.32* | 0.74* | -0.11 | 0.06 | 0.13 | -0.14* | 0.26* | 0.00 | 0.29* |
| 27. Blaming others W3 | 0.11 | 0.20* | 0.03 | 0.04 | -0.02 | 0.69* | 0.12 | 0.24* | 0.18* | 0.20* | 0.22* | 0.05 |
| 28. Internalizing problems W1 | -0.00 | -0.07 | 0.07 | 0.31* | 0.12 | 0.04 | -0.09 | -0.07 | -0.13 | -0.12 | -0.12 | 0.06 |
| 29. Internalizing problems W2 | 0.08 | -0.05 | 0.09 | 0.26* | 0.06 | 0.07 | -0.12 | -0.02 | -0.11 | 0.03 | -0.09 | 0.16* |
| 30. Internalizing problems W3 | 0.03 | -0.07 | 0.08 | 0.22* | 0.07 | 0.18* | -0.12 | -0.05 | -0.16* | 0.01 | -0.12 | 0.10 |
|  | 25. | 26. | 27. | 28. | 29. |  |  |  |  |  |  |  |
| 26. Self-blame W3 | 0.35* |  |  |  |  |  |  |  |  |  |  |  |
| 27. Blaming others W3 | 0.14* | -0.00 |  |  |  |  |  |  |  |  |  |  |
| 28. Internalizing problems W1 | 0.24* | 0.10 | 0.00 |  |  |  |  |  |  |  |  |  |
| 29. Internalizing problems W2 | 0.22* | 0.05 | 0.05 | 0.79* |  |  |  |  |  |  |  |  |
| 30. Internalizing problems W3 | 0.22* | 0.05 | 0.07 | 0.76* | 0.76* |  |  |  |  |  |  |  |

**Table S3***LPA fit for random subsample 1 (N = 252)*

|  |  |  |  |  |  | Percentage in each profile | | | | |
| --- | --- | --- | --- | --- | --- | --- | --- | --- | --- | --- |
|  | BIC | SSA-BIC | VLMR-LRT *p* | Entropy | ACPMLC | 1 | 2 | 3 | 4 | 5 |
| *Wave 1* | |  |  |  |  |  |  |  |  |  |
| 1 | 6350.51 | 6293.45 |  |  |  | 1.00 |  |  |  |  |
| 2 | 6234.85 | 6146.10 | .031 | .87 | .97 | .77 | .24 |  |  |  |
| 3 | 6151.00 | 6030.54 | .206 | .80 | .92 | .59 | .09 | .31 |  |  |
| 4 | 6114.92 | 5962.76 | .296 | .83 | .93 | .13 | .26 | .52 | .08 |  |
| 5 | 6110.91 | 5927.06 | .090 | .84 | .91 | .48 | .24 | .06 | .16 | .07 |
| *Wave 2* | |  |  |  |  |  |  |  |  |  |
| 1 | 2125.18 | 2068.38 |  |  |  | 1.00 |  |  |  |  |
| 2 | 2095.85 | 2007.49 | .347 | .78 | .94 | .67 | .33 |  |  |  |
| 3 | 2097.19 | 1977.27 | .165 | .83 | .95 | .55 | .24 | .22 |  |  |
| 4 | 2113.39 | 1961.92 | .532 | .88 | .95 | .55 | .23 | .15 | .08 |  |
| 5 | 2126.50 | 1943.48 | .690 | .90 | .98 | .06 | .15 | .51 | .20 | .08 |
| *Wave 3* | |  |  |  |  |  |  |  |  |  |
| 1 | 2772.29 | 2715.42 |  |  |  | 1.00 |  |  |  |  |
| 2 | .2737.31 | 2648.85 | .038 | .75 | .91 | .42 | .58 |  |  |  |
| 3 | 2709.43 | 2589.37 | .058 | .84 | .95 | .41 | .19 | .41 |  |  |
| 4 | 2727.48 | 2575.83 | .396 | .85 | .93 | .39 | .11 | .32 | .18 |  |
| 5 | 2742.96 | 2559.72 | .218 | .88 | .89 | .12 | .39 | .01 | .18 | .30 |

**Table S4***LPTA fit for random subsample 1*

| Number of profiles | Invariance | BIC | SSA-BIC | *LL* | *SCF* | *χ*^2^ (df) | *p* |
| --- | --- | --- | --- | --- | --- | --- | --- |
| 2 | Full | 10950.99 | 10792.48 | -5337.26 | 1.32 |  |  |
| 3 | Full | 10782.19 | 10566.62 | -5203.10 | 1.22 | 284.77(18) | < .001 |
| 4 | Full | 10762.03 | 10476.71 | -5132.19 | 1.17 | 139.66(22) | < .001 |
| 3 | Non-invariant | 10979.71 | 10592.96 | -5152.56 | 1.08 | 111.85(54) | < .001 |

*Note. N* = 252.

**Table S5**

*Description of the three profiles of the LPTA for random subsample 1*

|  | Proportion | | |  | |  | |  | Unstandardized mean score | | |  |  |  |
| --- | --- | --- | --- | --- | --- | --- | --- | --- | --- | --- | --- | --- | --- | --- |
|  | Wave 1 | Wave 2 | Wave 3 | Acceptance | Putting into perspective | | Positive refocusing | | Refocus on planning | Positive reappraisal | Rumination | Catastro-phizing | Self-blame | Blaming others |
| High adaptive,  low maladaptive | 32.6 | 23.1 | 44.3 | 3.51 | 3.18 | | 3.68 | | 3.45 | 3.43 | 2.95 | 1.68 | 2.35 | 1.96 |
| Low adaptive, high maladaptive | 44.3 | 23.8 | 23 | 2.76 | 2.62 | | 2.68 | | 3.14 | 2.53 | 3.67 | 3.66 | 3.27 | 1.94 |
| Generally low | 27.3 | 20.4 | 52.3 | 2.65 | 2.39 | | 2.78 | | 2.32 | 2.22 | 2.2 | 1.69 | 1.94 | 1.67 |

*Note. N* = 252.

**Figure S1**

*Visualization of the three LPTA profiles for random subsample 1*

**Table S6***LPA fit for random subsample 2 (N = 250)*

|  |  |  |  |  |  | Percentage in each profile | | | | |
| --- | --- | --- | --- | --- | --- | --- | --- | --- | --- | --- |
|  | BIC | SSA-BIC | VLMR-LRT *p* | Entropy | ACPMLC | 1 | 2 | 3 | 4 | 5 |
| *Wave 1* | |  |  |  |  |  |  |  |  |  |
| 1 | 6614.69 | 6557.64 |  |  |  | 1.00 |  |  |  |  |
| 2 | 6440.42 | 6351.66 | < .001 | .77 | .94 | .65 | .35 |  |  |  |
| 3 | 6402.20 | 6281.75 | .164 | .78 | .91 | .53 | .18 | .29 |  |  |
| 4 | 6336.79 | 6184.64 | .086 | .83 | .90 | .45 | .25 | .26 | .04 |  |
| 5 | 6330.65 | 6146.80 | .444 | .86 | .91 | .47 | .05 | .22 | .23 | .04 |
| *Wave 2* | |  |  |  |  |  |  |  |  |  |
| 1 | 2210.18 | 2153.38 |  |  |  | 1.00 |  |  |  |  |
| 2 | 2163.96 | 2075.60 | .029 | .80 | .97 | .52 | .48 |  |  |  |
| 3 | 2142.03 | 2022.11 | .187 | .84 | .94 | .44 | .16 | .40 |  |  |
| 4 | 2152.56 | 2001.08 | .243 | .88 | .92 | .16 | .39 | .37 | .08 |  |
| 5 | 2165.24 | 1982.21 | .434 | .92 | .94 | .37 | .03 | .37 | .16 | .07 |
| *Wave 3* | |  |  |  |  |  |  |  |  |  |
| 1 | 2867.80 | 2810.92 |  |  |  | 1.00 |  |  |  |  |
| 2 | 2771.76 | 2683.30 | .004 | .95 | .99 | .77 | .23 |  |  |  |
| 3 | 2756.30 | 2636.24 | .450 | .85 | .98 | .20 | .60 | .21 |  |  |
| 4 | 2746.39 | 2594.73 | .167 | .93 | .95 | .64 | .19 | .10 | .07 |  |
| 5 | 2761.78 | 2578.53 | .489 | .93 | .96 | .63 | .13 | .12 | .07 | .05 |

**Table S7***LPTA fit for random subsample 2*

| Number of profiles | Invariance | BIC | SSA-BIC | *LL* | *SCF* | *χ*^2^ (df) | *p* |
| --- | --- | --- | --- | --- | --- | --- | --- |
| 2 | Full | 11290.63 | 11132.12 | -5507.28 | 1.17 |  |  |
| 3 | Full | 11088.02 | 10872.46 | -5356.28 | 1.20 | 235.32(18) | < .001 |
| 4 | Full | 11027.26 | 10741.95 | -5265.16 | 1.14 | 190.92(22) | < .001 |
| 3 | Non-invariant | 11303.62 | 10916.87 | -5315.00 | 1.12 | 81.00(54) | .010 |

*Note. N* = 250.

**Table S8**

*Description of the three profiles of the LPTA for random subsample 2*

|  | Proportion | | |  | |  | |  | Unstandardized mean score | | |  |  |  |
| --- | --- | --- | --- | --- | --- | --- | --- | --- | --- | --- | --- | --- | --- | --- |
|  | Wave 1 | Wave 2 | Wave 3 | Acceptance | Putting into perspective | | Positive refocusing | | Refocus on planning | Positive reappraisal | Rumination | Catastro-phizing | Self-blame | Blaming others |
| Low adaptive, high maladaptive | 16.2 | 16.7 | 19.9 | 2.39 | 1.86 | | 1.96 | | 2.20 | 2.01 | 3.66 | 3.86 | 2.72 | 1.63 |
| Generally low | 49.9 | 48.4 | 50.5 | 2.77 | 2.37 | | 2.9 | | 2.29 | 2.27 | 2.08 | 1.63 | 1.87 | 1.66 |
| High adaptive,  low maladaptive | 33.9 | 34.9 | 29.6 | 3.37 | 3.52 | | 3.72 | | 3.61 | 3.35 | 2.99 | 2.16 | 2.53 | 2.01 |

*Note. N* = 250.

**Figure S2**

*Visualization of the three LPTA profiles for random subsample 2*

**Table S9**

*Cross-sectional associations between cognitive emotion regulation strategy profiles and internalizing problems at each timepoint, for the regular education subsample*

|  | *b* | β | *p* |
| --- | --- | --- | --- |
| **Wave 1** |  |  |  |
| Gender (girl) | 0.00 | -0.001 | .994 |
| High adaptive, low maladaptive | 0.01 | 0.02 | .720 |
| Low adaptive, high maladaptive | 0.11 | 0.20 | .002 |
| **Wave 2** |  |  |  |
| Gender (girl) | -0.03 | -0.11 | .249 |
| High adaptive, low maladaptive | 0.05 | 0.14 | .023 |
| Low adaptive, high maladaptive | 0.09 | 0.16 | .013 |
| **Wave 3** |  |  |  |
| Gender (girl) | 0.01 | 0.05 | .593 |
| High adaptive, low maladaptive | 0.01 | 0.03 | .676 |
| Low adaptive, high maladaptive | -0.003 | -0.01 | .930 |

*Note.* Reference group was “Generally low” profile (profile 1). For gender, boys were the reference group.

**References**

Achenbach, T. M., & Rescorla, L. A. (2001). *Manual for the ASEBA school-age forms &*

*profiles.* Burlington, VT: University of Vermont, Research Center for Children, Youth & Families.

Chen, F. F. (2007). Sensitivity of goodness of fit indices to lack of measurement invariance. *Structural Equation Modelling: A Multidisciplinary Journal, 14*, 464–504. <https://doi.org/10.1080/10705510701301834>

Little, T. D., Rhemtulla, M., Gibson, K., & Schoemann, A. M. (2013). Why the items versus parcels controversy needn’t be one. *Psychological Methods, 18*, 285–300. <https://psycnet.apa.org/doi/10.1037/a0033266>
